# D3 Receptor-Targeted Cariprazine: Insights from Lab to Bedside

**DOI:** 10.3390/ijms25115682

**Published:** 2024-05-23

**Authors:** Ágota Barabássy, Zsófia Borbála Dombi, György Németh

**Affiliations:** Medical Division, Gedeon Richter Plc., 1103 Budapest, Hungary; barabassya@richter.hu (Á.B.);

**Keywords:** cariprazine, D3 receptor, partial agonist, drug development, clinical development program

## Abstract

Until the late 1800s, drug development was a chance finding based on observations and repeated trials and errors. Today, drug development must go through many iterations and tests to ensure it is safe, potent, and effective. This process is a long and costly endeavor, with many pitfalls and hurdles. The aim of the present review article is to explore what is needed for a molecule to move from the researcher bench to the patients’ bedside, presented from an industry perspective through the development program of cariprazine. Cariprazine is a relatively novel antipsychotic medication, approved for the treatment of schizophrenia, bipolar mania, bipolar depression, and major depression as an add-on. It is a D3-preferring D3-D2 partial agonist with the highest binding to the D3 receptors compared to all other antipsychotics. Based on the example of cariprazine, there are several key factors that are needed for a molecule to move from the researcher bench to the patients’ bedside, such as targeting an unmet medical need, having a novel mechanism of action, and a smart implementation of development plans.

## 1. Introduction

Drug discovery and development dates back to the early days of human civilization, where medicines were derived mainly from plants and supplemented by animal materials and minerals [1]. These medicines were discovered through a combination of trial and error and observation of human and animal reactions after ingestion [1].

By the beginning of the 1900s, there were only a few medicines available for treating diseases, e.g., digitalis for cardiac conditions, quinine for malaria, ipecacuanha for dysentery, aspirin for fever, and mercury for syphilis [2]. The need for a ‘more systematic’ research emerged, and therefore the pharmaceutical industry was founded [2]. By 1930, drug discovery concentrated on isolating the active ingredients from natural products; later, these ingredients were synthesized, and the resulting products were called new chemical entities (NCEs) [2]. Many of the drugs synthesized were originally designed to treat other indications, and their final designation was still the matter of chance finding [2]. The discovery of chlorpromazine as an antipsychotic, for example, is often described as ‘serendipitous’, as it was originally synthesized for nausea and allergies; however, it was observed that it induced calmness when given to patients [3]. Other examples include the anxiolytic meprobamate, which was first used as an antimicrobial therapy [4], or the MAOI antidepressants that were first tried in tuberculosis [5].

Today’s drug development must go through many iterations and tests to ensure that the end product is safe, potent, and effective. It is a long and costly endeavor, with many pitfalls and hurdles. Only a few molecules make it into the clinical trial phase (only 1 molecule out of 5000–10,000) and even fewer will be marketed as actual medication [6]. This is especially true for psychiatric drugs, where the lines are even blurrier due to overlapping symptoms, receptor targets, and genetic mutations. In addition, the outcome parameters are soft, meaning that they are based on neuropsychiatric scales and tests that are subject to personal experience and thus subjectivity [7]. Successes can change the world, but failures are an inevitable part of the discovery and development process. However, in the end, if a medicine gets through the development process, it can clearly improve the life of millions. 

The aim of the present review is to provide an overarching insight into drug development from an industry perspective, using the example of cariprazine. Cariprazine is a rather new antipsychotic medication which was developed by Gedeon Richter Plc. It was granted marketing authorization by the US Food and Drug Administration (FDA) in 2015 under the brand name Vraylar [8] and by the European Medicines Agency (EMA) in 2017 under the brand name Reagila [9]. Subsequently, it was approved by several other national authorities globally; as of writing this review, it is prescribed to ca. 1.3 million patients suffering from different psychiatric disorders, including schizophrenia, dipolar mania, bipolar depression, and major depression (as an add-on) [8,9,10]. Drug development for cariprazine took more than 20 years, with various challenges and pitfalls in all stages of the development until the final approval by regulatory authorities and the treatment of patients. This review summarizes insights from previously published, company-sponsored, preclinical and clinical trials as available by its manufacturer, Gedeon Richter.

## 2. The Stages of Drug Development

**Figure 1:**
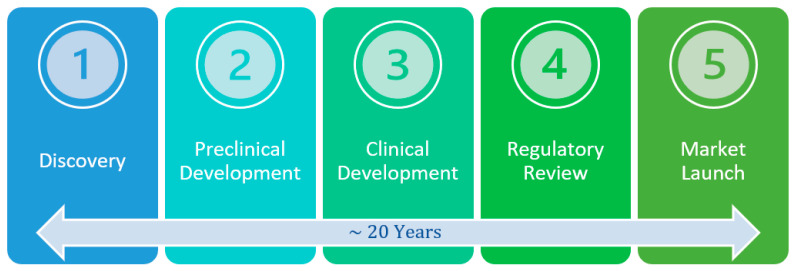
Stages of drug development.

Development and commercialization of a new molecule into a drug has five successive, interdependent stages (as shown in Figure 1 below) [11]. Successful drug development requires exceptional performance at each stage.

### 2.1. Molecule Discovery

In principle, there are two main directions to discover a molecule. First, when a compound is developed and then is researched to understand what kind of disorder it can successfully treat (the push strategy). Second, when unmet medical needs are defined in advance and the research focuses on developing specific compounds that can address these disorders (the pull strategy) [12]. Independent of the applied strategy, the new compound must differentiate from other existing drugs, either by addressing an unmet medical need or by providing better efficacy in partially improved patients [13]. 

As for cariprazine, targeted antipsychotic research was focused on the potential clinical effects of targeting D3 receptors in addition to classical dopamine D2 receptor antagonism or partial agonism. The potential importance of the D3 receptors in the treatment of psychiatric disorders has been examined since the early 1990s; pharmacological data were accumulating, and their relevance in neurological disorders was increasingly investigated [14], though, at that point, therapeutic potentials were not exploited yet. In Gedeon Richter Plc., ongoing research with dopamine D3 receptor selectivity lasted 20 years and laid the foundation for the birth of cariprazine [15]. The objective of the project was to develop a dopamine D3-preferring compound with sufficient partial agonist activity at the D2 receptors that would allow unmet medical needs to be addressed through its new biological target. 

Although there are some antipsychotics that leverage on other receptor targets [16,17], most antipsychotics available today target the D2 receptors, either as antagonists [18,19,20] or partial agonists [21]. The role of dopamine D2 receptors in the treatment of the positive symptoms of schizophrenia has been well established and forms the basis of schizophrenia treatment today [22]. However, approximately 30% of individuals diagnosed with schizophrenia experience symptoms that do not respond well to antipsychotic medications or only show minimal improvement [23,24]. In addition, a broad range of schizophrenia symptoms, including negative symptoms, or cognitive impairments remain largely unaffected by most antipsychotic treatments [25,26,27,28]. Antipsychotics may actually worsen or trigger negative and cognitive symptoms [25,26,27,28]. Unfortunately, these symptoms are closely linked to long-term disability in schizophrenia patients, making them a critical area of unmet medical need in clinical management [29,30]. 

The cariprazine clinical trial program [31,32] started at a point when the majority of antipsychotics on the market were primarily for the treatment of schizophrenia, and while there were already a sufficient number of treatments available for positive symptoms, there were no adequate therapeutic options for negative, affective, and cognitive ones. Additionally, there were also unmet medical needs in other major psychiatric disorders with similar symptoms to schizophrenia, such as bipolar disorder, major depression, post-traumatic stress syndrome, and certain neurological conditions. 

With cariprazine, the aim was to develop a D3-preferring D2-D3 partial agonist antipsychotics that might address a wide range of symptoms, including the negative and cognitive symptoms of schizophrenia and the affective symptoms in schizophrenia, next to generally addressing the symptoms of bipolar disorder and major depressive disorder. Additionally, there was a general need to reduce the side effects compared to existing drugs. 

These reasons combined have led scientists to look into the D3 receptor target. Dopamine D3 receptors are highly expressed in the ventral tegmental area, which is a group of dopaminergic cells that project to limbic structures like the nucleus accumbens [33,34,35,36,37,38,39,40]. In this area, the D3 receptors function as autoreceptors on the dopamine-producing cells themselves [33,34,35,36,37,38,39,40]. Additionally, D3 receptors are found postsynaptically at glutamatergic synapses within the nucleus accumbens, which is part of the limbic system, and presynaptically on the pyramidal cells in the fifth layer of the cortex, where they regulate the activity of the axon initial segment [33,34,35,36,37,38,39,40]. D3 receptors are involved in several CNS functions such as movement control, cognition, learning, reward, emotional regulation, and social behavior [38,39,40]. Stimulation of the D3 receptor produces a variety of effects, including negative symptom control in schizophrenia, cognitive symptom control, inhibition of locomotor activity, modulation of the self-administration of cocaine, and modulation of the self-stimulation of the ventral tegmental area [38,39,40].

Cariprazine is a D3-prefering D3-D2 partial agonsist antipsychotic. It occupies dopamine (D3/D2) receptors in the substantia nigra and ventral tegmental area and also in the ventral striatum (a part of the limbic system), as revealed by positron emission tomography using a dopamine D3-preferring agonist radiotracer, [11C](+)-PHNO [41]. The therapeutic effect of cariprazine is mediated through a combination of partial agonist activity at dopamine D3, D2 (Ki values of 0.085–0.3 nM and 0.49–0.71 nM, respectively), and serotonin 5-HT1A receptors (Ki values of 1.4–2.6 nM), and antagonist activity at serotonin 5-HT2B, 5-HT2A, and histamine H1 receptors (Ki values of 0.58–1.1 nM, 18.8 nM, and 23.3 nM, respectively) [9,42]. Cariprazine has a low affinity for serotonin 5-HT2C and adrenergic α1 receptors (Ki values of 134 nM and 155 nM, respectively) [9,42]. Cariprazine has no appreciable affinity for cholinergic muscarinic receptors (IC50 > 1000 nM) [9,42]. 

A PET study by Girgis et al. in 2016 found that administering 1 mg and 3 mg of cariprazine daily for two weeks to schizophrenia patients led to dopamine D2 receptor occupancies of 45% and 79%, respectively, and D3 receptor occupancies of 76% and 92% [41]. The study utilized positron emission tomography (PET) with the dopamine D3/D2 agonist radioligand 11C-PHNO to examine the occupancy of dopamine D3 and D2 receptors in the brains of schizophrenic patients aged 18 to 55 years [41]. Based on these findings, it is projected that daily doses of 4.5 and 6 mg of cariprazine would achieve full occupancy, particularly at D3 receptors [41].

Further, the ‘in vitro’ receptor kinetic parameters (Kd, Kon, and Koff) of cariprazine and didesmethyl cariprazine (DDCAR) in comparison with aripiprazole, haloperidol, clozapine, quetiapine, risperidone, and dopamine for human recombinant dopamine D2 and D3 was determined using the dopamine D2/D3 receptor antagonist [3H]raclopride as a radioligand according to the method of Sykes et al. 2010 [43]. Kon represents the rate constant of the association of a drug to its receptor, while Koff represents the rate constant of the dissociation of the drug from the receptor. Together, they describe the dynamic equilibrium between drug–receptor binding and unbinding. The Dissociation Constant (Kd) is the equilibrium constant for the association and dissociation of a receptor–ligand complex. Mathematically, it is defined as the ratio of koff to kon: Kd = koff/kon. A smaller Kd value indicates stronger binding affinity between the receptor and ligand. These are presented in Table 1 below.

Among the tested antipsychotics, both cariprazine and DDCAR showed the highest affinity and selectivity for dopamine D3 receptors (Kdkin values). Further, both cariprazine and DDCAR demonstrate a relatively slow association rate at dopamine D2 and D3 receptors compared to the investigated antipsychotics (Kon values). Additionally, data suggest that cariprazine is unique among antipsychotics for its high affinity for the D3 receptor, surpassing even dopamine itself, which typically has a strong natural affinity for this receptor [41,44,45,46,47]. While other antipsychotics show higher ‘in vitro’ affinity for the D3 receptor than dopamine, cariprazine stands out with an affinity approximately a thousand times greater [41,44,45,46,47]. This significant difference suggests that cariprazine may be one of the few drugs capable of effectively binding to the D3 receptor in the presence of dopamine (in the living brain) when used to treat psychosis [41,44,45,46,47]. The Koff data indicate that, under ‘in vitro’ conditions, both cariprazine and its major human metabolite, DDCAR, showed a relatively slow dissociation rate (i.e., a relatively long residence time) at both D2 and D3 receptors. Aripiprazole demonstrated the slowest dissociation, whereas clozapine and quetiapine readily dissociated from these receptors.

In addition to its unique pharmacological properties, cariprazine stands out from other antipsychotics due to its exceptionally long half-life [9]. It also has two main active metabolites, desmethyl cariprazine and didesmethyl cariprazine, which exhibit receptor-binding and functional activity similar to the parent drug [9]. After stopping or pausing the dosage, the plasma levels of total cariprazine decrease slowly [9]. The concentration is halved in approximately one week and reduces by more than 90% within about three weeks [9]. 

### 2.2. Drug Development—Preclinical and Clinical

Once a compound is synthesized and deemed appropriate, it enters the preclinical phase. In this phase, hypotheses of efficacy are examined in animal models. If signs of efficacy are seen, further studies are conducted in different species of animals to test for the potential molecule’s safety parameters. If these studies are successful, the chosen drug candidate requires a patent application covering the molecule at the world’s largest pharmaceutical markets, most importantly, the US, the European Union, and Japan. This is to ensure that the subsequent development program that costs of billions of dollars is provided exclusivity. 

If a molecule successfully passes the animal studies, it enters the clinical phase. The decision to start a clinical development program implies a commitment to carry out different stages of development. The cost of running a full clinical trial program for new molecules ranges from USD 161 million to USD 4.54 billion (2019 USD) [48] and requires considerable logistical preparation from conceptual design to operational feasibility. The financial backing for such a development must be secured. Phase III trials are often run on several continents, involving thousands of patients in multiple hospital centers. Today, only multinational companies are able to finance global drug development on such a scale, or smaller companies if they find a partner to co-develop globally. 

A study examining the progress of thousands of development programs from 2006–2015 found that the cumulative chance of a new molecule progressing from a successful Phase I program to Phase II and then to Phase III, followed by FDA approval, was just 6.2%; meaning that, on average, 15 out of 16 clinical programs fail before reaching their target [49]. A more recent report [11] shows that the success rates have improved slightly. According to a report by Deloitte, the probability of a drug candidate progressing from Phase I to FDA approval increased to 10% for the period of 2010–2019. However, it is essential to note that drug development remains a challenging and costly process, with many candidates still failing to reach the market. This is also true for antipsychotics [50]. Over the past decade (2011–2020), only a few new antipsychotic medications have been introduced, including cariprazine, brexpiprazole, lumetaperon, and pimavanserin, for the treatment of psychosis in dementia [50]. Pimavanserin failed to demonstrate efficacy in schizophrenia negative symptoms [51], and another promising compound, roluperidone, for the treatment of negative symptoms in patients with schizophrenia also failed approval [50,52]. Moreover, there is a huge hurdle for the probability of success (PoS) of an approval by the FDA for the first indication, whereas subsequent indications and approvals by other regulatory authorities after initial approval by the FDA are easier. This poses several difficult questions, such as which disorder to target, and which development has the highest probability of success and return of investment.

The first stage, most commonly performed on healthy volunteers, is the Phase I trial, which is designed to assess the safety and tolerability [53,54,55]. These trials involve a small cohort of 20 to 80 healthy volunteers. The goal is to determine the highest dose humans can tolerate without serious side effects. Unlike later phases, Phase I participants are generally healthy individuals, and exclusion criteria ensure that they have no significant health issues. The duration of Phase I trials spans several months, during which escalating doses are administered to understand the drug’s impact and identify any adverse effects. Key activities include studying pharmacokinetics (PKs) and pharmacodynamics (PDs), which provide insights into the drug’s effects on the body [53,54,55]. 

Following the Phase I studies, during Phase II clinical trials, the aim is to assess both the effectiveness and safety of an investigational drug. The study participants, numbering several hundred, represent the real-world patient population for whom the drug is intended. Key activities include dose optimization, exploring different dosages to identify the most effective and safe regimen, and exploratory trials, which delve into secondary endpoints and potential mechanisms of action. Based on the results, the drug faces a critical decision point: proceed to Phase III or discontinue further development. Approximately 70% of medications advance to Phase III after successful Phase II trials; so, Phase II serves as a crucial crossroad, inching researchers closer to transforming scientific promise into tangible patient benefits [53,54].

The first Phase II proof-of-concept studies with cariprazine were performed in the indications of schizophrenia and mania. Most of the antipsychotics on the market at the time were first developed for the schizophrenia indications, followed by an indication expansion if relevant. Considering the novel mechanism of action and potential added benefits of cariprazine also in mood disorders, a different development approach was decided: parallel exploratory Phase II trials in two indications (schizophrenia and mania). 

Both proof-of-concept studies (schizophrenia and mania) clearly demonstrated the efficacy of cariprazine over the placebo in a dose-dependent manner [56,57]. Additionally, the studies showed that cariprazine had beneficial effects on certain symptom clusters in schizophrenia [58] (negative, cognitive, and affective), clusters that are also present in other psychiatric disorders, e.g., bipolar depression and major depression. However, these initial proof-of-concept studies were not designed to demonstrate effects on these subdomains/disorders and, therefore, the decision had to be made whether to carry out studies examining efficacy in the subdomains of schizophrenia or efficacy in other indications. 

An additional aspect to consider is that regulatory pathways in the US are different compared to Europe. While the FDA only requires short-term studies in combination with long-term safety data to approve an indication such as schizophrenia or mania, the EMA requires additional long-term maintenance-of-effect studies. Therefore, different approaches need to be pursued in different regions. As for cariprazine, in the US, studies in other indications were prioritized, which investigated the efficacy of cariprazine in the treatment of mania, bipolar depression, and major depressive disorder as an add-on treatment (aMDD). Studies have shown the superiority over the placebo for 3.0–6.0 mg cariprazine in mania and 1.5–3.0 mg cariprazine in bipolar depression and aMDD, based on which the FDA has granted approval in these additional three indications [10]. 

In Europe, considering that further costly, long-term, maintenance-of-effect studies were required for the approval of these indications, the decision was taken to rather focus on subpopulations within schizophrenia where an unmet medical need was present. Based on the existing EMA-issued regulatory guideline for schizophrenia, a specifically designed study to examine cariprazine’s efficacy in the persistent, predominant, primary negative symptoms of schizophrenia was conducted and compared to the standard-of-care treatment (risperidone) [32]. The study concluded that cariprazine was more effective than risperidone in improving the negative symptoms in patients with schizophrenia, suggesting that it could be a more beneficial treatment option for this condition [32].

There are also various pitfalls in drug development, such as non-conclusive studies, adverse events, and authority rejections. Non-conclusive, failed studies are the biggest fear in drug development, as you invest a lot of money in vain. This is partly due to the fact that, as per regulatory requirements, the used statistical methods account for the risk of false-negative outcomes, and therefore yield negative results even for effective drugs. Looking at compounds which have achieved regulatory approval, about half of the trials failed to demonstrate efficacy using the regulatory-requested statistical methods [59]. Processing these studies, drawing conclusions, and adjusting the development strategy and the design of further trials are key steps in the successful implementation of an overall program. Additionally, narrow segments of indications, such as subpopulations or areas of unmet needs, should be more in focus rather than covering a whole range of diverse symptoms and illness phases. 

All medicines have side effects, some more severe ones, some less severe. The key issue is not to be without side effects, but for the benefits to outweigh the risks (risk–benefit ratio). The adverse reaction profile of a drug has the following two extremes: 1. the most unfavorable outcome is when a development program needs to be stopped due to a serious adverse reaction which threatens the health and wellbeing of patients; 2. the most favorable outcome, on the other hand, is when a compound not only has better efficacy, but also better safety compared to competitors. This can be a differentiating (competitive) advantage for the compound.

### 2.3. Regulatory Review

The FDA is the gatekeeper of the world’s largest pharmaceutical market and gateway to other highly regulated markets [60]. All major pharmaceutical players try to register their compounds with the FDA. Their new drug application review process can end in one of two ways: 1. the approval of the compound; 2. the complete response letter, which is equivalent to a rejection. In the context of first-time drug applications, several factors contribute to unsuccessful outcomes with the FDA. A large-scale study showed that 15.9% of applications faced uncertainties related to dose selection, while 13.2% encountered challenges with study endpoints that did not adequately reflect clinically meaningful effects [61]. Additionally, inconsistent results emerged when different endpoints were tested (13.2%) or when comparing trials conducted at different sites (11.3%) [61]. Furthermore, 13.2% of drugs demonstrated poor efficacy when compared to the standard of care [61].

As for cariprazine, it was granted marketing authorization by the FDA in 2015 under the brand name Vraylar [8] and by the EMA in 2017 under the brand name Reagila [9]. 

### 2.4. Pharmaceutical Market Launches and Post-Authorization Activities

The last phase of drug development is market entry and medical product management. In terms of the time horizon, there is a significant overlap between running the clinical studies and market introduction due to post-authorization studies, clinical studies in different indications, and different approval processes globally. 

Launching medicines into global markets is a complex and multifaceted process that involves strategic planning, regulatory compliance, market analysis, and effective distribution channels [62]. Companies must establish a clear vision for their international business [62]. Decisions on whether to enter a market directly or through alliances, copromotion, or licensing partnerships are crucial [62]. Strategic context, long-term goals, and the potential impact on existing markets and pipeline value are important factors to consider [62]. Navigating the regulatory landscape is essential [62]. Each country has its own regulatory body and set of requirements for drug approval [62]. Understanding and adhering to these regulations is vital for a successful launch [62]. Additionally, understanding the local epidemiology, treatment paradigms, and patient needs is key to determining the potential success of a medicine [62]. Companies must analyze the market to tailor their product positioning and marketing strategies accordingly [62]. Establishing efficient distribution systems is also critical to ensure that medicines are accessible to healthcare providers and patients [62]. This may involve setting up local subsidiaries or working with local distributors [62]. Setting an appropriate price point and ensuring patient access to medicines are also important [62]. This includes negotiating with payers and health authorities to secure reimbursement [62]. Effective marketing strategies are needed to raise awareness and encourage the adoption of the new medicine [62]. This often involves educational campaigns for both healthcare professionals and patients [62]. After launch, companies must monitor the performance of their product, gather real-world evidence, and make adjustments to their strategies as needed [62].

While clinical trials with cariprazine in bipolar depression and major depressive disorder still continued, in 2017, the EMA granted a centralized marketing authorization (across all EU countries) for the schizophrenia indication [9]. For this approval, the EMA requested data on both the short- and long-term efficacy of cariprazine in patients suffering from schizophrenia, which required the completion of a 92-week study [9]. In contrast, in the US, long-term efficacy data had to be submitted as a post-approval commitment only. Further, based on the positive outcome of three additional Phase III studies in patients with bipolar depression, the FDA granted marketing authorization in 2019 [8]. In line with its requirements, it requested additional long-term studies as a post-approval commitment. Thereafter, in December 2022, the FDA further approved cariprazine for the treatment of major depressive disorder as an add-on treatment [10]. 

After market access, the next big challenge is to inform the medical community. This needs to be performed in a factual, balanced manner through publications, posters, conference communications, standalone events, scientific advisory board meetings, and local language regional events. For cariprazine, the number of international publications is well over 100, including highly reputable journals such as *The Lancet*. There are more than 400 posters and over 700 oral presentations at major international events have been performed. Medical information services and online platforms (e.g., https://schizophrenia.life/ (accessed on 16 May 2024)) were put in place to inform the medical community about cariprazine and its clinical data.

It is through the medical community that the information reaches the patients, the end users of any treatment. Cariprazine is the fastest growing oral antipsychotic in the US. Globally, around 1 M patients have been treated with the drug and more than 4 million prescriptions have been filled since its launch in March 2016.

## 3. Summary and Conclusions

The aim of this review was to provide an overarching insight into drug development from an industry perspective. It identified a few factors that are needed for a molecule to move from the researcher bench to the patients’ bedside, which are summarized in Table 2.

However, this review is not without limitations. Firstly, drug development was only presented for the example of cariprazine. Although there have not been many successful new drug developments in the field of antipsychotics in the past years, one development (lumetaperone [63]) might further finetune the conclusions drawn. Secondly, as drug development is a lengthy endeavor spanning potentially 20 years, where regulatory requirements, unmet needs, and competitor landscapes might change, future perspectives and conclusions are subject to change also. Finally, interpretation might be confounded by the nature of the work, mainly focusing on previously published, company-sponsored, preclinical and clinical trials as available by the manufacturer, Gedeon Richter.

The drug development of cariprazine has been a classic drug development, going through all the stages from the research lab to Phase I, II, and III trials. Although regulatory guidelines still expect the same clinical trial programs and have changed only little in the past years [64,65], there are trends for the future that will accelerate the process of development and improve patient outcomes. One of the key trends that have already been recognized by regulatory authorities is using real-world data and real-world evidence (RWE). The FDA created a framework in 2018 for evaluating the potential use of RWE to help support the approval of a new indication for a drug already approved or to help support or satisfy drug post-approval study requirements [66]. Other key trends include artificial intelligence (AI) and data analytics. AI can handle vast amounts of data, aiding in target identification, big data analytics, patient matching, and automating molecule design. It has the potential to significantly reduce the time required to develop new drugs [67]. Other trends include the following [68]:

1. A shift toward patient-centric trials that ensure that patients’ needs and experiences are at the forefront. This approach enhances trial design, recruitment, and overall drug development efficiency.
2. Advances in assay technologies, which enable better understanding of drug mechanisms and efficacy, leading to more precise drug development.
3. Innovations in manufacturing processes, which enhance the scalability, quality, and cost-effectiveness of drug production.
4. Leveraging synthetic biology, which allows for the creation of novel molecules and pathways, expanding the drug development toolbox.
5. Virtual trials, conducted remotely with digital tools, which reduce costs and streamline data collection, making drug development more efficient.
6. Three-dimensional cell culture, mimicking human tissue environments using 3D cell culture systems, which provides more accurate drug testing and reduces the reliance on animal models.

Collectively, these innovations aim to expedite drug development, improve safety, and bring effective therapies to patients faster, and are the future of drug development.

## Figures and Tables

**Table 1:** ‘In vitro’ receptor kinetic parameters (Kdkin, Kon, and Koff) of cariprazine and didesmethyl cariprazine (DDCAR) relative to aripiprazole, haloperidol, clozapine, quetiapine, risperidone, and dopamine.

	Kdkin Values (nM) (Affinity; the Smaller the Number, the Higher the Affinity)	Kon (min^−1^ nM^−1^) (Association; the Smaller the Number, the Faster the Association Rate)	Koff (min^−1^) (Dissociation Rate; the Smaller the Number, the Lower the Dissociation Rate)
D2 receptors	DDCAR (0.21) > aripiprazole (0.60) > cariprazine (0.69) > haloperidol (0.95) > risperidone (1.1) >> clozapine (27) > quetiapine (79) >> dopamine (701)	dopamine (0.0002) >> quetiapine (0.007) > aripiprazole (0.009) > clozapine (0.028) > cariprazine (0.058) > risperidone (0.158) > DDCAR (0.531)	aripiprazole (0.006) < cariprazine (0.032) < DDCAR (0.104) = dopamine (0.105) < risperidone (0.172) < haloperidol (0.438) < quetiapine (0.535) < clozapine (0.816)
D3 receptors	DDCAR (0.11) > cariprazine (0.17) > haloperidol (0.95) > aripiprazole (3.9) > risperidone (5.9) > dopamine (21) >> clozapine (90) > quetiapine (163)	quetiapine (0.001) = aripiprazole (0.001) > dopamine (0.003) > clozapine (0.007) > cariprazine (0.034) ~ risperidone (0.042) > haloperidol (0.072) >> DDCAR (0.165)	aripiprazole (0.005) < cariprazine (0.006) < DDCAR (0.015) < haloperidol (0.066) < dopamine (0.077) < risperidone (0.201) < quetiapine (0.22) < clozapine (0.677)

**Table 2:** Factors of a successful drug development.

1	Targeting an unmet medical need.
2	Developing a drug with a novel mechanism of action.
3	Exceptional and flexible performance in each development stage, considering risk/benefit and cost/benefit principles.
4	Perseverance and commitment to follow through despite obstacles.
5	Prioritizing high probability of success pathways.
6	Creativity in identifying and evaluating opportunities and overcoming pitfalls.
7	Analyses of potential—later/earlier discovery, how many similar drugs are on the market, what development projects are ongoing in parallel, the degree of market saturation, and the patent status of other drugs.
8	Forward and reverse translation—next to translation from animal results to clinical data, the utilization of patient data to update subsequent development programs.

## Data Availability

No new data were created or analyzed in this study. Data sharing is not applicable to this article.
